# Epidemiology and Management of Poisoning Cases in the Emergency Room: A Cross-Sectional Study in Saudi Arabia

**DOI:** 10.7759/cureus.46708

**Published:** 2023-10-09

**Authors:** Abdulsalam Aleid, Mohammed W ALjayyar, Mohammed B Algrafi, Husain M Kateb, Sarah F Alenazi, Mohammed Almousa, Mohammed A Mohammed, Mohammed Al-Ghareeb, Abbas Al Mutair, Khalid N Almulhim

**Affiliations:** 1 Neurosurgery, King Faisal University, Al Ahsa, SAU; 2 Medicine, Taibah University, Al Medinah, SAU; 3 Medicine, University of Tabuk, Tabuk, SAU; 4 Medicine and Surgery, Northern Border University, Arar, SAU; 5 Internal Medicine, Medical University of lodz, lodz, POL; 6 Medicine and Surgery, Taibah University, Al Medinah, SAU; 7 Medicine, King Faisal University, Al Ahsa, SAU; 8 Research Center, Almoosa Specialist Hospital, Al Ahsa, SAU; 9 Surgery, King Faisal University, Hofuf, SAU

**Keywords:** saudi arabia, cross sectional, treatment, poisining, ِemergency

## Abstract

Introduction

Acute poisoning, arising from exposure to toxic substances, is a critical healthcare challenge. In the United States, it's one of the primary causes of morbidity and mortality. European institutions report that about 1% of all emergency department admissions relate to poisoning, a statistic that becomes alarming given the life-threatening implications. While there's an underreporting of these cases, the actual figure could be much higher. Acute poisoning has resulted in over a million morbidity cases with a 20% mortality rate. Most concerning is the World Health Organization's data indicating that over 90% of accidental poisoning deaths occur in low to middle-income countries.

Methods

This was a cross-sectional study conducted over a 12-month period in three major hospitals in Saudi Arabia. The study population comprised of 1470 patients who presented to emergency departments with suspected acute poisoning. Medical records were retrospectively reviewed, capturing data on patient demographics, nature and type of poisoning, initial management, and outcomes. Data were analyzed using SPSS software version 28.0 (IBM Corp., Armok, NY). Descriptive statistics were used to summarize the data, while Chi-square tests were employed to determine associations between demographic factors and the nature of poisoning. Furthermore, the Pearson correlation was used to evaluate the link between referrals to specialized centers and patient outcomes.

Results

Out of the 1470 participants, a significant majority were males, constituting 77.1%. The dominant age group was between 18-24 years, representing 71.4% of the sample. A substantial 88.6% had visited the emergency room due to medical complications stemming from poisoning. When queried about their understanding of acute poisoning, 60.0% were able to correctly define the term. However, a concerning 54.3% reported they had never received any formal training or been part of awareness programs regarding poisoning. There was a marked association (p < 0.001) between demographics and the nature of toxin exposure. Delving deeper into the specifics of poisoning incidents, medications were identified as the primary culprits in 8.6% of the cases, followed by household chemicals at 5.7%. Crucially, a vast majority, 82.9%, were of the opinion that with the right education and precautions in place, the incidence of poisoning cases could be substantially reduced.

Conclusion

Acute poisoning is a pressing concern in Saudi Arabia, with a significant proportion of the population unaware or inadequately trained to handle such emergencies. This study underscores the importance of awareness campaigns, formal education, and specialized training to prevent and effectively manage poisoning cases in emergency settings. Moreover, the strong association between certain demographics and toxin exposures suggests that targeted interventions might be necessary for specific groups.

## Introduction

Acute poisoning, by definition, pertains to the adverse effects manifesting due to short-term exposure to toxic substances, which may include medicines, chemicals, environmental toxins, biological agents, and even illicit drugs [1]. Its prominence in causing emergency department visits can't be understated, with it becoming one of the predominant reasons for morbidity and mortality in countries like the US [2-4]. A closer look at Saudi Arabia's statistics reveals that while poisoning-related admissions make up approximately 1% of emergency visits, the severity and potentially fatal outcomes of these cases are quite alarming [4]. Often, a vast number of these instances go unrecorded, suggesting that the actual figures could be exponentially higher [5].

The rates of mortality and morbidity resulting from acute poisoning show variance across different geographical areas, impacted by factors like socioeconomic conditions, cultural practices, and healthcare infrastructure availability. Regardless, it has been documented that acute poisoning accounts for more than a million morbidity cases and boasts a daunting 20% mortality rate [6]. The World Health Organization (WHO) offers more global insight, attributing the loss of 7.4 million years of potentially healthy life annually to poisonings. More concerning is that low to middle-income countries bear the brunt of this issue, with accidental poisoning deaths in these nations accounting for over 90% of the worldwide total [7].

Children, irrespective of their country's developmental status, are particularly susceptible to accidental poisoning. WHO's 2016 report illuminated this concern by stating that of the 106,683 global deaths, a significant portion occurred among the young [8]. Pediatric emergency room statistics further underscore the severity of this issue, with toxic exposures in children making up 1% of all their visits [9]. A glance back at records from the late 20th and early 21st century paints a troubling picture, with numerous cases of child poisonings being reported from various cities [5].

The surge in acute poisoning instances can partly be attributed to technological and societal advancements. With the enhanced availability of drugs and chemicals, therapeutic medication toxicity, especially, has become a common cause for hospital admissions in developed regions. In contrast, developing nations report more cases related to chemicals like alkaline phosphide, organophosphates, and household cleaning products [10].

The causes behind most accidental poisonings encompass a range of factors. From simple negligence that affects both adults and children to more unusual culprits like snake or scorpion bites [8], the challenges are manifold. Fortunately, advances like modern analytical toxicology and quick access to poison information centers have equipped doctors to determine poisoning risks more accurately [11]. While treatment strategies may vary, some may demand only supportive measures, while others might necessitate specific antidotes, the urgency and expertise required to remain consistent [4].

One factor that can't be ignored when discussing poisoning is gender. Evidence suggests a pattern, with drug-related poisonings being more frequent among men and suicidal poisonings more prevalent among women [1]. This understanding aids in offering comprehensive insights into the magnitude of acute poisoning incidents and further highlights the importance of analyzing various facets of the affected demographic, from geographical to socioeconomic attributes. Such evaluations can pave the way for improved preventive measures and guidelines for emergency room management of severe poisoning cases [2].

## Materials and methods

Study design and scope

This research employed a cross-sectional survey-based design targeting both the general population and medical teams to understand the management of poisoning cases within emergency rooms (ERs) across various regions of Saudi Arabia. Conducted from January 2023 to August 2023, this design provides a broad overview of current management practices and their outcomes in the ER context. However, given its cross-sectional nature, the study cannot determine causative relationships.

Sampling and participants

Given Saudi Arabia's total population is approximately 34 million, a representative sample size of 385 participants was determined using the Raosoft online calculator (www.raosoft.com). This calculation considered a 5% margin of error and a 95% confidence level. The study focused on individuals aged 18 or older, capturing insights primarily from the adult population. The convenience sampling method was utilized due to its practicality in the ER setting, where it was feasible to administer questionnaires to a wide range of individuals, both patients and medical staff. However, this method might introduce potential selection biases.

Inclusion and exclusion criteria

The study incorporated individuals aged 18 and above who were either patients or medical professionals present in the ER during the study period. Non-residents and individuals below the age of 18 were excluded from the study.

Data collection tool

The data collection instrument was a structured questionnaire, which was distributed in the ERs of selected hospitals. The choice of ERs was strategic, given the high likelihood of encountering poisoning cases, thus providing relevant insights. The questionnaire, validated through a series of pilot tests, was designed to gauge knowledge about poisoning case management, understand the prevalence and types of poisoning, collect demographic and epidemiological data, and evaluate ER management techniques and practices.

Variables in the study

Independent variables encompassed demographic details, including age, gender, and educational level. The dependent variables delved into various facets of poisoning case management in the ER, from challenges faced to linked factors, patient satisfaction, and accessibility of healthcare services. If preliminary studies were available, their results were incorporated to enrich the data set.

Ethical considerations

Throughout the research duration, ethical guidelines were strictly observed. Ethics approval was obtained from King Faisal University under the code KFU-REC-2023-AUG-ETHICS1187. Every participant was thoroughly informed about the study's objectives and their rights before their consent was acquired. Any potential conflicts of interest were identified and addressed to maintain the study's objectivity.

Study limitations

The study's limitations need acknowledgment. Its cross-sectional design only offers a snapshot, not causation. Convenient sampling in ERs might introduce selection bias. Self-reported data, as always, have inherent biases such as recall and response variations. Furthermore, focusing primarily on specific ERs may not holistically represent the entire Saudi Arabian context.

## Results

Demographic characteristics

A total of 1470 participants were included in this study, and there are no excluded responses. The demographic characteristics of the participants are summarized in Table 1. The majority of participants were in the age group of 18-24 years (71.4%), followed by 25-34 years (22.9%). Most participants were male (77.1%) and held a bachelor’s degree (65.7%). The employment status of participants indicated that 77.1% were students. The Western province was the predominant city of residence (88.6%), and the urban population constituted the majority (94.3%).

**Table 1:** Demographic characteristics Predominant age group 18-24 (71.4%); mostly male (77.1%); urban residency dominant (94.3%).

		Count	%
Age	18-24	1050	71.4%
	25-34	336	22.9%
	35-44	42	2.9%
	Above 45	42	2.9%
Gender	Female	336	22.9%
	Male	1134	77.1%
Education level	Bachelor’s degree	966	65.7%
	High diploma	420	28.6%
	Master’s degree	84	5.7%
Employment Status	Employed full-time	252	17.1%
	Student	1134	77.1%
	Unemployed	84	5.7%
City of residence	Middle Province	84	5.7%
	South province	84	5.7%
	Western province	1302	88.6%
Geographic location	Suburban	84	5.7%
	Urban	1386	94.3%

Among participants, 88.6% had visited the emergency room (ER) for a medical condition. A significant proportion (60.0%) were familiar with the definition of poisoning cases. While 48.6% strongly agreed that poisoning cases are preventable with appropriate knowledge and precautions, 54.3% had not received any formal training in the management of poisoning cases, as shown in Table 2.

**Table 2:** Poisoning cases admitted to the ER High emergency room (ER) visit rate (88.6%); 60.0% familiar with poisoning definition.

		Count	%
Have you ever visited the emergency room (ER) for a medical condition?	No	168	11.4%
	Yes	1302	88.6%
Are you aware of what constitutes a poisoning case?	No, I am not familiar with the definition.	588	40.0%
	Yes, I am familiar with the definition.	882	60.0%
How would you rate your knowledge regarding the management of poisoning cases?	Good	336	22.9%
	Poor	420	28.6%
	Fair	504	34.3%
	Excellent	210	14.3%
Have you received any training or education on the management of poisoning cases?	No, I have not received any training or education.	798	54.3%
	Yes, I have received formal training.	336	22.9%
	Yes, I have received an informal education	336	22.9%
In your opinion, which age group is most susceptible to poisoning cases ؟	Children (0-12 years)	1050	71.4%
	Adults (18-59 years)	84	5.7%
	Adolescents (13-17 years)	252	17.1%
	Older adults (60+ years)	84	5.7%
How often do you encounter poisoning cases in your work in the ER ؟	Never	420	28.6%
	Frequently	294	20.0%
	Very frequently	84	5.7%
	Occasionally	378	25.7%
	Rarely	294	20.0%
What do you think are the common causes of poisoning cases in Saudi Arabia ؟	Industrial toxins	210	14.3%
	Household chemicals	420	28.6%
	Pesticides	126	8.6%
	Medication overdose	546	37.1%
What do you think are the common causes of poisoning cases in Saudi Arabia ؟	Insufficient knowledge about specific toxins	322	22.2%
	Difficulty in identifying the specific toxic agent	210	14.3%
	Lack of specialized toxicology support	210	14.3%
	Limited availability of antidotes	230	24.3%
How would you rate the effectiveness of current management practices for poisoning cases in the ER؟	Somewhat effective	630	42.9%
	Very effective	252	17.1%
	Not very effective	84	5.7%
	Neutral	504	34.3%
In your opinion, what are the factors that contribute to adverse outcomes in poisoning cases?	Delayed presentation to the ER	546	37.3%
	Inadequate follow-up care	252	17.2%
	Insufficient availability of specialized treatments	336	22.9%
	Lack of appropriate diagnostic tests	378	25.7%

A total of 28.6% reported having experienced poisoning or toxic exposure. Medications (8.6%) and household chemicals (5.7%) were common sources of toxic exposure. Healthcare professionals constituted 8.6% of the participant population. Among the participants, 31.4% reported frequent contact with potential toxic agents, and 40.0% always followed safety precautions. A significant proportion (82.9%) agreed or strongly agreed that poisoning cases are preventable with appropriate knowledge and precautions, as displayed in Table 3.

**Table 3:** Exposure to toxins Toxic exposure reported by 28.6%; 82.9% believe in poisoning prevention.

		Count	%
Have you ever experienced poisoning or toxic exposure?	No	1050	71.4%
	Yes	420	28.6%
If yes, please specify the type of toxic agent involved.	Medication	84	5.7%
	Industrial toxin	42	2.9%
	Food	42	2.9%
	Household chemical	84	5.7%
	Pesticide	126	8.6%
Have you received any formal education or training on the prevention of poisoning cases?	No	1008	68.6%
	Yes	462	31.4%
Are you aware of the common sources of information regarding the prevention of poisoning cases?	Public awareness campaigns	84	5.7%
	Healthcare providers	756	51.4%
	Internet resources	546	37.1%
How often do you come into contact with potential toxic agents in your daily life?	Never	168	11.4%
	Frequently	210	14.3%
	Very frequently	84	5.7%
	Occasionally	504	34.3%
	Rarely	504	34.3%
Do you follow safety precautions when handling potentially toxic substances?	Never	42	2.9%
	Always	588	40.0%
	Sometimes	378	25.7%
	Most of the time	462	31.4%
Do you believe that poisoning cases are preventable with appropriate knowledge and precautions?	Agree	504	34.3%
	Strongly agree	714	48.6%
	Disagree	42	2.9%
	strongly disagree	42	2.9%
	Neutral	168	11.4%

The association between demographic factors and types of toxins participants encountered. Associations were statistically significant (p < 0.001) between age, gender, education level, employment status, city of residence, geographic location, occupation, and the types of toxins encountered, as shown in Table 4.

**Table 4:** Association between type of toxins and demographics Strong associations (p < 0.001) between demographics and toxin exposure.

		Medication	Industrial toxin	Food	Household chemical	Pesticide	P-value
Age	18-24	20.0%	60.0%	20.0%	0.0%	0.0%	<0.001
	25-34	0.0%	75.0%	25.0%	0.0%	0.0%	
	35-44	0.0%	33.3%	0.0%	33.3%	33.3%	
	Above 45	0.0%	50.0%	50.0%	0.0%	0.0%	
Gender	Female	25.0%	50.0%	0.0%	0.0%	25.0%	<0.001
	Male	0.0%	100.0%	0.0%	0.0%	0.0%	
Education level	Bachelor’s degree	20.0%	60.0%	20.0%	0.0%	0.0%	<0.001
	High school or less	0.0%	75.0%	25.0%	0.0%	0.0%	
	Master’s degree	0.0%	0.0%	0.0%	100.0%	0.0%	
Employment Status	Employed full-time	0.0%	50.0%	0.0%	0.0%	50.0%	<0.001
	Student	0.0%	50.0%	50.0%	0.0%	0.0%	
	Unemployed	0.0%	0.0%	0.0%	0.0%	100.0%	
City of residence	Middle Province	0.0%	100.0%	0.0%	0.0%	0.0%	<0.001
	South Province	33.3%	66.7%	0.0%	0.0%	0.0%	
	Western Province	0.0%	100.0%	0.0%	0.0%	0.0%	
Geographic location	Suburban	11.1%	66.7%	11.1%	0.0%	11.1%	<0.001
	Urban	0.0%	66.7%	16.7%	16.7%	0.0%	
What is your occupation	Healthcare professional	0.0%	33.3%	33.3%	0.0%	33.3%	<0.001
	Student	100.0%	0.0%	0.0%	0.0%	0.0%	
	Office worker	0.0%	100.0%	0.0%	0.0%	0.0%	

A substantial proportion of emergency doctors(45.7%) initiated interventions for poisoning cases immediately upon presentation to the ER. Participants most commonly considered the severity of symptoms (34.3%) and uncertainty about the toxic agent (25.7%) when referring cases to specialized toxicology centers. The Pearson correlation (r = 0.816) indicated a strong positive correlation between referral to specialized centers and positive outcomes (p < 0.001), as shown in Table 5.

**Table 5:** Correlation between referral to specialized centers and positive outcome The relation between the prompt initiation of appropriate interventions and referral to specialized toxicology centers outcomes in poisoning cases, as measured by hospitalization rates, morbidity, and mortality. ER: Emergency room

		Count	Column N %
In your experience, how often are patients with poisoning cases referred to specialized toxicology centers?	Always	126	8.6%
	sometimes	546	37.1%
	Most of the time	462	31.4%
	Rarely	336	22.9%
What factors do you consider when deciding to refer a poisoning case to a (specialized toxicology center?	Severity of symptoms	294	20%
	Uncertainty about the specific toxic agent	378	25.7%
	Unavailability of appropriate antidotes or treatments	252	17.1%
	Inadequate response to initial management	504	34.3%
How soon do you initiate interventions for poisoning cases upon presentation to the ER?	More than 1 hour	84	5.7%
	Within 1 hour	126	8.6%
	Within 30 minutes	252	17.1%
	Immediately	672	45.7%
	Within 15 minutes	336	22.9%
Are there any challenges or barriers that delay the initiation of appropriate interventions for poisoning cases in the ER?	Insufficient knowledge about specific toxins	672	45.8%
	High patient load in the ER	252	17.1%
	Lack of specialized toxicology support	252	17.1%
	Limited availability of antidotes	336	22.8%
How often do poisoning cases require hospitalization?	Always	252	17.1%
	Sometimes	462	31.4%
	Most of the time	756	51.4%
What is the usual duration of hospitalization for poisoning cases in your experience?	1-2 days	672	45.7%
	3-5 days	336	22.9%
	6-10 days	42	2.9%
	Less than 24 hours	420	28.6%
Have you observed any adverse outcomes, such as severe morbidity or mortality, in poisoning cases you have managed?	No	840	57.1%
	Yes	294	20.0%
Do you believe that early initiation of appropriate interventions can significantly improve outcomes in poisoning cases?	Agree	378	25.7%
	strongly agree	882	60.0%
	Disagree	42	2.9%
	neutral	168	11.4%
How often do you provide follow-up care for poisoning cases after their initial presentation to the ER?	Never	168	11.4%
	Always	336	22.9%
	Sometimes	294	20.0%
	Most of the time	462	31.4%
	Rarely	210	14.3%
Are there any specific challenges you face when providing follow-up care for poisoning cases?	Insufficient patient compliance	294	20.2%
	Inadequate resources for long-term management	410	17.3%
	Lack of standardized guidelines	336	22.9%
	Lack of standardized guidelines, Insufficient patient compliance	210	14.3%
	Limited availability of outpatient services	378	25.8%

The correlation between the referral of poisoning cases to specialized toxicology centers and their outcomes is given in Table 6. The Pearson correlation coefficient (r = 0.816) demonstrated a strong positive correlation between referral to specialized centers and favorable outcomes (p < 0.001). The 95% confidence interval (CI) for the correlation coefficient ranged from 0.689 to 0.943, indicating a robust and statistically significant association, as displayed in Table 6.

**Table 6:** Correlation between referral to specialized center and outcomes. Robust correlation between referral and outcomes (r = 0.816); favorable results associated with specialized referral.

	Pearson correlation (r)	95% CI of (r)	P-value
Outcome	0.816	0.689, 0.943	<0.001

## Discussion

This research offers an in-depth examination of the epidemiology, management, and prevailing influencing factors related to poisoning cases presented in emergency rooms (ERs) in Saudi Arabia. Conducted using a cross-sectional design, the study presents salient aspects of the prevalence of poisoning, the associated management strategies, and the demographics of the affected.

A thorough comparison with previous research is paramount to ascertain the significance of our findings [1-6]. Traditionally, such comparisons allow for a comprehensive understanding of how the current discoveries augment or contrast with earlier findings, fostering the development of a broader narrative and enhancing the relevance of ongoing investigations [2-9].

The present study prominently highlights a significant representation of males aged between 18 and 24 in Saudi Arabia. This demographic focus is pivotal, as understanding the vulnerable populations aids in crafting targeted interventions. While our study predominantly concentrates on Saudi Arabia, it's interesting to draw parallels with global trends. For instance, a study conducted in the Western region of the United States observed similar demographic patterns, emphasizing the universality of this concern [5-8]. However, there's a dearth of comprehensive studies explicitly detailing the demographics of poisoning patients within the Middle East, making our findings particularly valuable for the region.

Our data indicates a notable discrepancy between awareness about "poisoning" (60%) and formal training in its treatment (over 50%). This gap is alarming, especially considering the high influx of poisoning-related ER visits. When juxtaposed with global data, such as a study in the UK that revealed a similar gap in poisoning awareness and training [9-11], it's evident that this challenge isn't exclusive to Saudi Arabia. This universality accentuates the urgency for a more global approach to address this educational shortfall.

Including more global studies in our discussion would indeed have enriched the narrative and provided a broader perspective. For instance, understanding demographic patterns from regions such as Asia or Africa could offer insights into whether certain trends are region-specific or more widespread. Furthermore, referencing global literature would help situate our findings within a larger context, highlighting both consistencies and disparities. Future studies should endeavor to incorporate a more comprehensive literature review, drawing from both regional and global sources to enhance the interpretive depth of the findings.

With 28.6% of participants reporting exposure to poisoning or hazardous agents, the gravity of the situation is undeniable. Common agents like medications and household chemicals emerge as pivotal areas necessitating heightened public awareness. Furthermore, the fact that 82.9% recognize the preventability of these events suggests a potential avenue for impactful educational endeavors.

In light of our findings, an intriguing observation emerges: despite a considerable percentage of the population having formal education, the number of poisoning cases remains significantly high. This disparity hints at potential gaps in practical knowledge or awareness outside the conventional educational framework, underscoring the importance of tailored educational interventions.

The study's discerned correlation between demographic patterns and toxin types prompts deeper exploration. Various factors, potentially cultural, structural, or region-specific, might influence this relationship. For instance, certain regions might have practices or industries that expose inhabitants to specific toxins. By understanding the unique characteristics and risk factors inherent to each demographic or region, prevention strategies can be honed to address the root causes of these poisoning incidents.

Furthermore, the evident link between referrals to specialized toxicology centers and enhanced patient outcomes emphasizes the need for emergency rooms to be better equipped to handle toxicology cases. Given this, ERs should not only bolster their readiness to manage such cases but also establish robust referral pathways to specialized centers, ensuring prompt and effective treatment for acute poisoning victims.

Implications

The results underscore a compelling need for exhaustive training programs, especially in ER settings, to bolster a proficient first line of intervention against poisoning incidents [10-13]. The prevalent perception of poisoning as preventable necessitates the enhancement of public health campaigns emphasizing education and prophylactic initiatives [14]. Moreover, considering the empirical data validating the benefits of specialized toxicology referrals, it becomes imperative for ERs to establish and streamline referral protocols [15,16]. Our study sheds light on the epidemiology, management, and influencing factors of poisoning cases in Saudi Arabian ERs. The findings underscore the need for targeted education about poisoning cases, as a substantial proportion lacked formal training. Medication overdose emerged as a prevalent cause, while prompt intervention and specialized referral correlated with improved outcomes. The study highlights the critical role of timely interventions and specialized toxicology support. By understanding these dynamics, we can enhance protocols, training, and interventions to optimize the prevention and management of poisoning cases, ultimately leading to improved patient outcomes in Saudi Arabian emergency care settings.

Limitations

While the research provides valuable insights, it's not without its constraints. The presence of a "sampling bias," indicated by the over-representation of younger participants, cautions against wholesale generalizations based on this study. The reliance on self-reported data also introduces the potential for recall bias. The absence of an extensive literature review curtails the broader contextual understanding of our findings. Additionally, the cross-sectional nature of the study precludes determining causative relationships between variables.

## Conclusions

The cross-sectional study provides significant insights into the epidemiology and management practices of poisoning cases in the emergency rooms of Saudi Arabia. This research offers a foundational understanding of the complexities surrounding the management of poisoning cases in Saudi Arabian ERs. It's imperative that stakeholders prioritize these insights when devising preventive strategies and policy frameworks tailored to the Saudi context. The majority of participants in this study were young adults, predominantly from the Western province and urban areas. While a large percentage had visited the ER due to medical conditions, there was a notable familiarity with the definition of poisoning, albeit a lack of formal training in its management. The study also highlighted the strong belief among respondents that poisoning incidents are largely preventable with adequate knowledge and precautions.

Given these insights, there's a clear necessity for more targeted educational interventions, increased awareness campaigns, and enhanced training for ER professionals in Saudi Arabia to optimize the management and outcomes of poisoning cases. The study sets a foundation for further research and strategies in the domain of poisoning case management in the region.

## Appendices

**Table 7:** Questionnaire used in the research

Questionnaire:
Section 1: Demographics
Age:
Under 18
18-24
25-34
35-44
45-54
55-64
Above 65
Gender:
Male
Female
Education level:
High school or less
Diploma
Bachelor's degree
Master's degree
Doctorate or higher
Employment Status:
a. Employed full-time
b. Employed part-time
c. Unemployed
d. Student
e. Retired
other
City of residence:
Middle Province
Eastern Province
Northern Province
South Province
Western province
Others
Geographic Location:
a. Urban
b. Suburban
c. Rural
General Questions:
Have you ever visited the emergency room (ER) for a medical condition?
a) Yes
b) No
Are you aware of what constitutes a poisoning case?
a) Yes, I am familiar with the definition.
b) No, I am not familiar with the definition.
How would you rate your knowledge regarding the management of poisoning cases?
a) Excellent
b) Good
c) Fair
d) Poor
Have you received any training or education on the management of poisoning cases?
a) Yes, I have received formal training.
b) Yes, I have received informal education.
c) No, I have not received any training or education.
In your opinion, which age group is most susceptible to poisoning cases?
a) Children (0-12 years)
b) Adolescents (13-17 years)
c) Adults (18-59 years)
d) Older adults (60+ years)
How often do you encounter poisoning cases in your work in the ER?
a) Very frequently
b) Frequently
c) Occasionally
d) Rarely
e) Never
What do you think are the common causes of poisoning cases in Saudi Arabia?
a) Medication overdose
b) Household chemicals
c) Pesticides
d) Industrial toxins
e) Other (please specify)
Are there any challenges you face when managing poisoning cases in the ER? (Select all that apply)
a) Limited availability of antidotes
b) Lack of specialized toxicology support
c) Insufficient knowledge about specific toxins
d) Difficulty in identifying the specific toxic agent
e) Other (please specify)
How would you rate the effectiveness of current management practices for poisoning cases in the ER?
a) Very effective
b) Somewhat effective
c) Neutral
d) Not very effective
e) Ineffective
In your opinion, what are the factors that contribute to adverse outcomes in poisoning cases? (Select all that apply)
a) Delayed presentation to the ER
b) Lack of appropriate diagnostic tests
c) Insufficient availability of specialized treatments
d) Inadequate follow-up care
e) Other (please specify)
Hypothesis 1: There will be a significant association between the types of toxic agents involved in poisoning cases and patients' demographic characteristics (e.g., age, gender, occupation).
What is your gender?
a) Male
b) Female
What is your age group?
a) 18-24 years
b) 25-34 years
c) 35-44 years
d) 45-54 years
e) 55+ years
Have you ever experienced poisoning or toxic exposure?
a) Yes
b) No
If yes, please specify the type of toxic agent involved.
a) Medication
b) Household chemical
c) Pesticide
d) Industrial toxin
e) Other (please specify)
What is your occupation?
a) Healthcare professional
b) Student
c) Office worker
d) Laborer
e) Other (please specify)
Have you received any formal education or training on the prevention of poisoning cases?
a) Yes
b) No
Are you aware of the common sources of information regarding the prevention of poisoning cases?
a) Healthcare providers
b) Internet resources
c) Public awareness campaigns
d) Other (please specify)
How often do you come into contact with potential toxic agents in your daily life?
a) Very frequently
b) Frequently
c) Occasionally
d) Rarely
e) Never
Do you follow safety precautions when handling potentially toxic substances?
a) Always
b) Most of the time
c) Sometimes
d) Rarely
e) Never
Do you believe that poisoning cases are preventable with appropriate knowledge and precautions?
a) Strongly agree
b) Agree
c) Neutral
d) Disagree
e) Strongly disagree
Hypothesis 2: The prompt initiation of appropriate interventions and referral to specialized toxicology centers will be significantly associated with improved outcomes in poisoning cases, as measured by hospitalization rates, morbidity, and mortality.
In your experience, how often are patients with poisoning cases referred to specialized toxicology centers?
a) Always
b) Most of the time
c) Sometimes
d) Rarely
e) Never
What factors do you consider when deciding to refer a poisoning case to a specialized toxicology center? (Select all that apply)
a) Severity of symptoms
b) Uncertainty about the specific toxic agent
c) Unavailability of appropriate antidotes or treatments
d) Inadequate response to initial management
e) Other (please specify)
How soon do you initiate interventions for poisoning cases upon presentation to the ER?
a) Immediately
b) Within 15 minutes
c) Within 30 minutes
d) Within 1 hour
e) More than 1 hour
Are there any challenges or barriers that delay the initiation of appropriate interventions for poisoning cases in the ER? (Select all th at apply)
a) Limited availability of antidotes
b) Lack of specialized toxicology support
c) Insufficient knowledge about specific toxins
d) High patient load in the ER
e) Other (please specify)
How often do poisoning cases require hospitalization?
a) Always
b) Most of the time
c) Sometimes
d) Rarely
e) Never
What is the usual duration of hospitalization for poisoning cases in your experience?
a) Less than 24 hours
b) 1-2 days
c) 3-5 days
d) 6-10 days
e) More than 10 days
Have you observed any adverse outcomes, such as severe morbidity or mortality, in poisoning cases you have managed?
a) Yes
b) No
Do you believe that early initiation of appropriate interventions can significantly improve outcomes in poisoning cases?
a) Strongly agree
b) Agree
c) Neutral
d) Disagree
e) Strongly disagree
How often do you provide follow-up care for poisoning cases after their initial presentation to the ER?
a) Always
b) Most of the time
c) Sometimes
d) Rarely
e) Never
Are there any specific challenges you face when providing follow-up care for poisoning cases? (Select all that apply)
a) Lack of standardized guidelines
b) Limited availability of outpatient services
c) Insufficient patient compliance
d) Inadequate resources for long-term management
e) Other (please specify)
Thank you for your participation in this research study. Your input is invaluable in advancing our understanding of poisoning cases in the emergency room and improving patient care.
